# T2 Dixon Imaging in the Evaluation of Hibernoma: Reliable Identification of Macroscopic Fat

**DOI:** 10.5334/jbsr.4037

**Published:** 2025-11-11

**Authors:** Sander Gurdeep Singh, Wouter Huysse, Frederiek Laloo

**Affiliations:** 1 UZ Gent, 9000 Gent, Belgium; 2 AZ Sint-Lucas, 9000 Gent, Belgium

**Keywords:** hibernoma, dixon

## Abstract

*Teaching point:* T2 Dixon imaging is valuable in assessing soft tissue lesions. Water-only images highlight fluid or edema, while fat-only images identify macroscopic fat and help delineate fat-devoid regions, which often indicate suspicious areas—or confirm their absence in benign lesions.

## Case

A 44-year-old woman was referred to the radiology department for chronic left-sided hip pain. No previous medical history was known.

**Figure 1:**
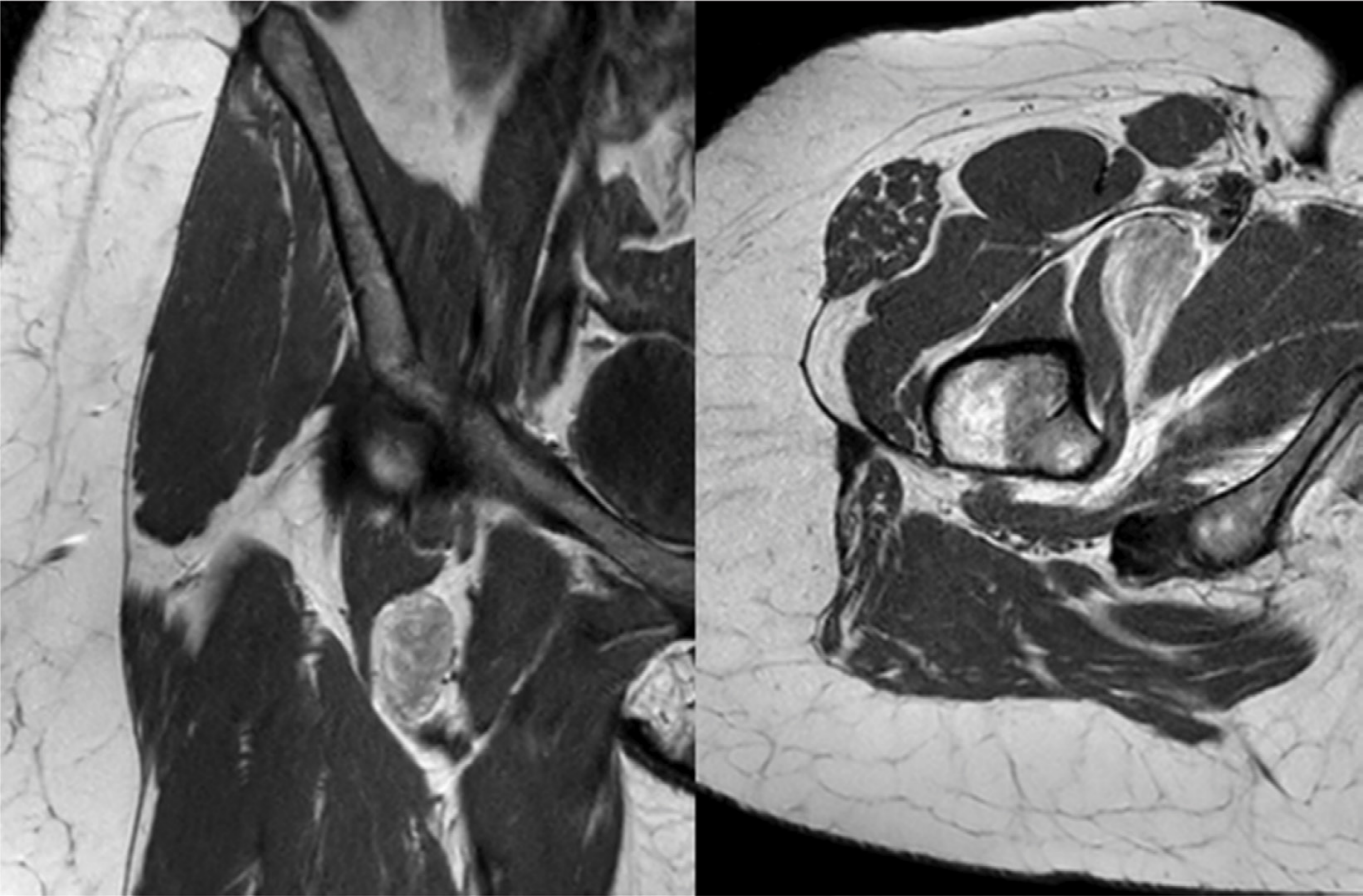
Coronal and axial T1 weighted MRI images revealed a well-circumscribed mass in the deep right inguinal region with mixed signal intensities.

Imaging of the left hip demonstrated marked joint space narrowing due to cartilage loss and rim geodes (not shown). Magnetic resonance imaging (MRI) revealed a well-circumscribed mass in the deep right inguinal region with mixed signal intensities on T1-weighted imaging (Figure 1).

**Figure 2:**
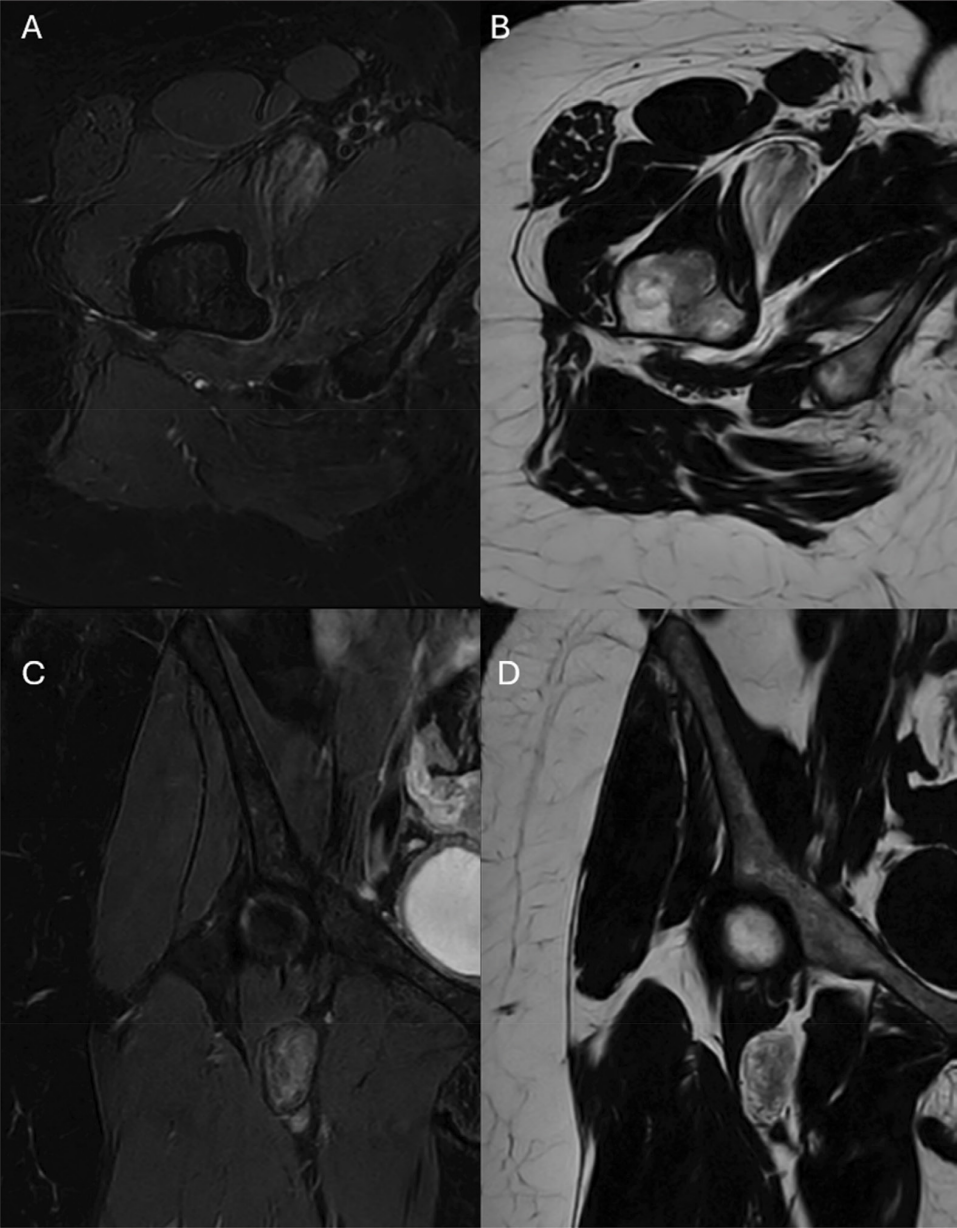
T2 Dixon MRI images demostrate the regions of high signal on the fat-only images **(B** and **D)** corresponding to low signal on the water-only images **(A** and **C)** with interspersed brush-stroke-like areas of high signal intensity on the water-only images and low signal on the fat-only images.

Further evaluation of the lesion was performed using T2 Dixon imaging in the axial and coronal planes. The lesion harbored regions of high signal on the fat-only images (Figure 2A and C) and with corresponding signal loss on the water-only images (Figure 2B and D), consistent with macroscopic fat. Interspersed within this fat-containing lesion were brush-stroke-like areas of high signal intensity on the water-only images and low signal on the fat-only images, indicating reduced adipose content. However, the absence of complete signal loss suggested that some macroscopic fat remained interspersed within these regions.

**Figure 3:**
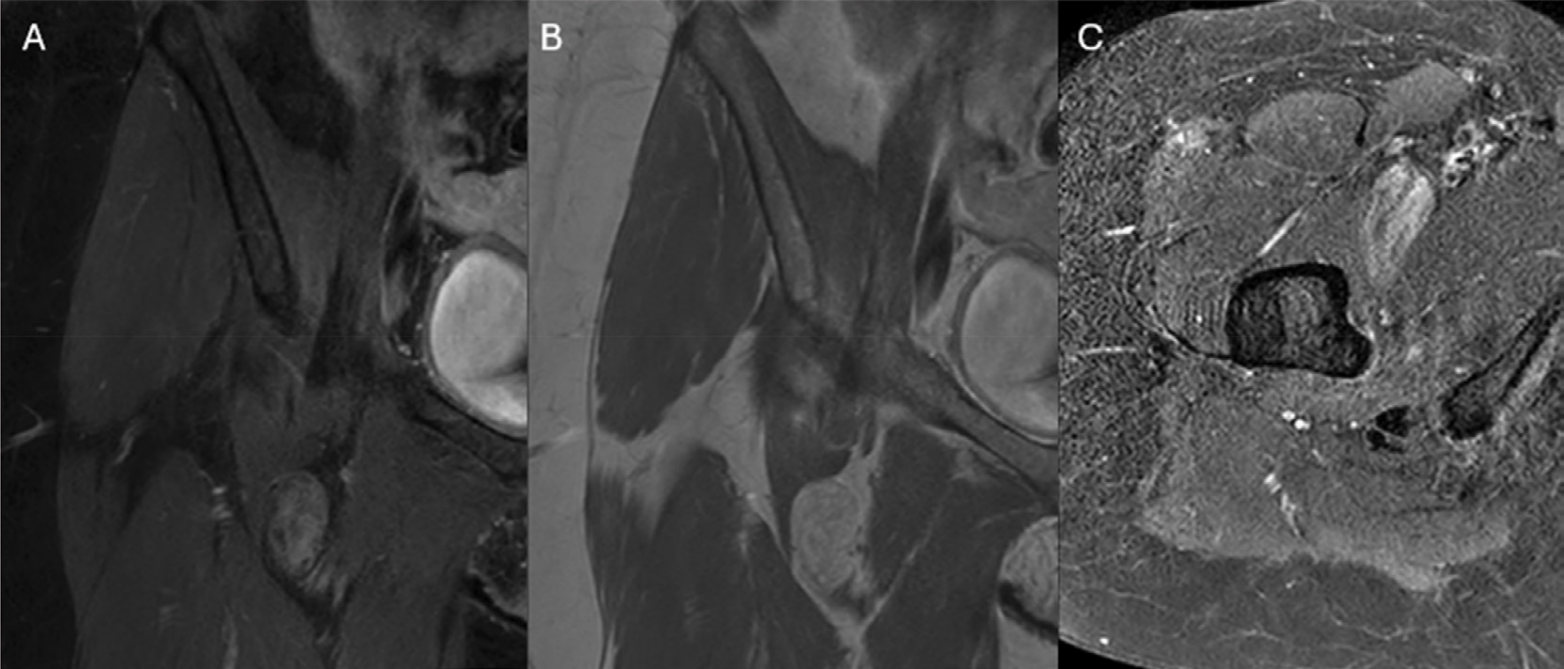
Coronal post-contrast enhanced images **(A** and **B)** and axial post-contrast SPIR images demonstrate enhancement of the regions of low fat-content.

Coronal contrast-enhanced T1 Dixon images (Figure 3A and B) and axial T1 spectral presaturation with inversion recovery (SPIR, Figure 3C) imaging demonstrated enhancement of these regions of (remnants of) low adipose tissue without involvement of the adjacent structures.

## Comment

Hibernomas are rare benign fatty tumors of fetal brown fat and refer to the resemblance of brown fat in hibernating animals. These tumors are most often seen in young adults with a slight male predominance. They tend to be located in regions where brown fat is typically present. Histology obviates the regions of brown fat interspersed with normal white adipocytes. Symptoms are often limited to a painless swelling of the soft tissue, and on rare occasions, they are due to mass effect [1].

These tumors are commonly biopsied and resected, but depending on the histological subtype, they may show benign features on MRI, more particularly the absence of nodular fat-devoid areas. T2 Dixon imaging is especially supportive in this context, as it confirms this absence through the lack of distinct areas of complete signal loss on fat-only images.

Hibernomas generally demonstrate heterogeneous contrast enhancement, reflecting fibrovascular tissue interspersed between areas of fat. Enhancement patterns may vary depending on the histological subtype. Correlation with T2 Dixon imaging can further support the presence of macroscopic fat between the enhancing fibrovascular components.
